# Animal Models of Tuberculosis Vaccine Research: An Important Component in the Fight against Tuberculosis

**DOI:** 10.1155/2020/4263079

**Published:** 2020-01-02

**Authors:** Wenping Gong, Yan Liang, Xueqiong Wu

**Affiliations:** Army Tuberculosis Prevention and Control Key Laboratory/Beijing Key Laboratory of New Techniques of Tuberculosis Diagnosis and Treatment, Institute for Tuberculosis Research, The 8 Medical Center of Chinese PLA General Hospital, 17 Heishanhu Road, Haidian District, Beijing 100091, China

## Abstract

Tuberculosis (TB), an infectious disease caused by *Mycobacterium tuberculosis*, is one of the top ten infectious diseases worldwide, and is the leading cause of morbidity from a single infectious agent. *M. tuberculosis* can cause infection in several species of animals in addition to humans as the natural hosts. Although animal models of TB disease cannot completely simulate the occurrence and development of human TB, they play an important role in studying the pathogenesis, immune responses, and pathological changes as well as for vaccine research. This review summarizes the commonly employed animal models, including mouse, guinea pig, rabbit, rat, goat, cattle, and nonhuman primates, and their characteristics as used in TB vaccine research, and provides a basis for selecting appropriate animal models according to specific research needs. Furthermore, some of the newest animal models used for TB vaccine research (such as humanized animal models, zebrafish, *Drosophila*, and amoeba) are introduced, and their characteristics and research progress are discussed.

## 1. Introduction

Tuberculosis (TB) is a major human infectious disease caused by a single organism, and was responsible for 1.6 million deaths, including human immunodeficiency virus (HIV)-associated TB deaths, with 10 million new TB cases diagnosed in 2017 worldwide [1]. The development of novel vaccines is considered a high priority in protecting human beings against TB disease worldwide. Currently, 22 new TB vaccines are being evaluated in clinical trials, four of which [Vaccae (*Mycobacterium vaccae* for injection) in patients with latent TB infection (LTBI), *Mycobacterium indicus pranii* (MIP)/Mw, Utilins (*Mycobacterium phlei*), and VPM1002 (rBCG *ΔureC::hly*)] have reached Phase III clinical trials [2–4]. Furthermore, three therapeutic vaccines [Vaccae, Utilins, and BCG Polysaccharide and Nucleic Acid Injection (BCG-PSN)] have obtained registration certificates from the China Food and Drug Administration (http://eng.sfda.gov.cn/WS03/CL0755/) and have been widely used to clinically treat TB in China [4]. In comparison with TB vaccines at the stage of clinical trials, there are many more vaccine candidates emerging in preclinical stages of development.

Promotion of the development of TB vaccines using humans as experimental subjects is fraught with challenges. Accumulation of clinical research is not only limited by time and space but also the several ethical and methodological restrictions of experiments with human subjects. The main advantage of an animal model is that it overcomes these deficiencies, and this essential role in the preclinical research of TB vaccines is receiving increasing attention. The superiority of using an animal model is mainly manifested in the following aspects: (1) the risks of experimentation on humans are avoided; (2) experimental conditions can be strictly controlled, and comparability of experimental materials is enhanced; (3) experimental operation and sample collection are simplified; and (4) a more comprehensive understanding of the nature of TB can be achieved.

Because of these advantages, various animal models have been generated for testing TB vaccines. However, the strategy of using animal models has begun to shift from an empirical-based approach to focus on the 3Rs principle (replacement, reduction, and refinement) [5]. Therefore, establishing methods to evaluate the immune protective efficiency and safety of TB vaccines using the smallest number of animals possible has become a scientific priority. Herein, we review the advantages and disadvantages of animal models, as well as clinical trials for TB vaccine research, and suggest that the goal of realizing a successful TB vaccine to the market stage is inseparable from the selection of appropriate animal models in preclinical testing.

## 2. Current Animal Models Used in TB Vaccine Research

**Figure 1:**
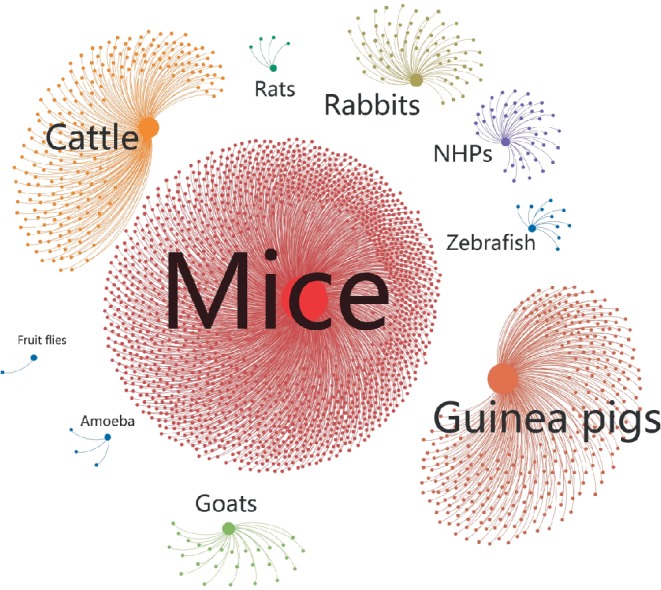
*Statistical map of the utilization of different animal models in preclinical studies of TB vaccines*. The source of the publications was an NCBI (National Center for Biotechnology Information) PubMed search using the keywords (vaccine AND tuberculosis AND ten categories shown in figure). The statistics were plotted using an open source graph visualization and manipulation software termed Gehpi. Each study is represented by a blue dot, and each animal model is represented by a circle of different color. The circle size represents the frequency of use of the animal model.

Animal models are not only valuable for understanding the humoral and cellular immune responses against *M. tuberculosis *but are also essential to evaluate the safety, immunogenicity, and protective efficacy of TB vaccine candidates. The main animal models used in TB vaccine research according to a search of the PubMed database are schematically presented in Figure 1 and listed in Table 1. Each of these animal models has its own characteristics that make it suitable for studying candidate TB vaccines; therefore, the choice and utilization of animal models should depend on the purpose of the experiment, availability of space, stage of the vaccine, financial resources, trained staff, laboratory conditions, and other available resources (Table 1). In addition, pathological characteristics are the consequence of host-pathogen interactions mediated by immunologic responses; thus, these features are directly relevant to the strengths and limitations of the different models used in evaluating vaccine candidates. Previous studies have suggested that classical granulomas with similarity to those in humans could be observed in guinea pig, rabbit, rat, nonhuman primate (NHP), cattle, and goat animal models, but not in common mouse, fruit fly, and amoeba animal models (Table 1). In general, small animal models are used for large-scale screening of TB vaccines, such as mice, guinea pigs, rabbits, and zebrafish, which are not only economical but also readily available. Once a vaccine with good protective efficacy has been identified, it can be further evaluated in large animal models such as NHPs, which, although expensive, can more closely mimic the immune responses of humans to reliably test the protective efficacy of the potential TB vaccine. Furthermore, these animal models play key roles in evaluating the safety of vaccines, including mice for acute toxicity and drug distribution, monkeys for chronic toxicity, guinea pigs for skin allergic reactions, and rabbits for skin irritation.

### 2.1. Small Mammalian Models

Small mammals are the most widely used type of animal models in preclinical studies of TB vaccines for several reasons, including easy operation, easy access, clear genetic background, low cost, easy feeding, and more abundant commercial reagents. The most profound advantage of these models is their cost-effectiveness, allowing for numerous applications and detailed characterization. However, small mammalian animal models differ from humans with respect to genetics and immunology. Therefore, such models, especially murine models, are more suitable for screening candidate vaccines for TB on a large scale.

#### 2.1.1. Mice

Mice have been the most widely used small animal model in the initial screening of TB vaccine candidates and for evaluating the efficacy of new vaccine candidates because of their low cost, rapid propagation, feasibility of use in the laboratory, long-term survival, mature immunological evaluation indices, and more abundant commercial reagents. The most popular mouse strains used for these purposes are BALB/c and C57BL/6, which both show variations in the susceptibility to infection of the *M. tuberculosis* H37Rv strain according to different challenge routes, with doses of tail vein injection, intraperitoneal injection, and aerosol attack of 1–5 × 10^5^ colony-forming units (CFUs), 1 × 10^6^ CFUs, and 0.5–1 × 10^2^ CFUs [43, 44], respectively. Both of these mouse strains also show equivalent protective efficacy for evaluating the Bacillus Calmette–Guérin (BCG) vaccine (the current clinically used TB vaccine) [45]. Moreover, the differences in animal models and immunization routes will affect the protective response induced by vaccines. Stylianou et al. [46] reported that when BALB/c and C57BL/6 mice were primed with BCG and boosted 10 weeks later with ChAdOx1.PPE15 vaccine, followed by challenge with aerosolized *M. tuberculosis*, the booster ChAdOx1.PPE15 only improved the protection provided by BCG in C57BL/6 mice and not in BALB/c mice. A recent study compared the effects of different immunization routes [intranasal (i.n.), subcutaneous (s.c.), and intramuscular (i.m.)] on immune responses against the recombinant protein ESAT-6/CFP-10 of *M. tuberculosis* in a mouse model, and found that the titers of specific antibodies were quickly elevated in s.c. and i.m. immunized mice compared to those in i.m. immunized mice, whereas the i.n. immunized mice showed lower levels of interleukin (IL)-5 production [47]. Some previous studies also suggested that the BCG vaccine could induce similar immune responses and protection by rectal and parenteral immunization routes in BALB/c mice [48]; s.c. and i.n./oral immunization with Ag85A-Mtb32 exhibited the strongest boosting effects for BCG-primed systemic and pulmonary cell-mediated immunity responses in C57BL/6 mice [49], respectively. These results highlight the importance of considering differences between mouse models as well as immunization routes when evaluating TB vaccine in mice.

Interestingly, a growing number of studies have suggested that immunization with most BCG or recombinant BCG (rBCG) vaccines could induce a significantly strong Th1-type immune response, characterized by enhanced IgG2a/IgG1, IgG2b/IgG1, or IgG2c/IgG1 ratios, as well as a high expression level of Th1 cytokines [interferon (IFN)-*γ*, tumor necrosis factor (TNF)-*α*, and IL-2) in C57BL/6 or BALB/c mouse models [50–57]. Additionally, a previous study reported that immunization of a new recombinant BCG vaccine, rBCG-CMX (composed of immune-dominant epitopes from Ag85C, MPT51, and HspX), could present higher amounts of Th1, Th17, and polyfunctional specific T cells in a murine model [58]. In contrast, a small number of BCG or rBCG vaccines led to a relatively high Th2 response, as evidenced by the high IgG1/IgG2a ratio and the low IFN-*γ* levels in these murine models [59–62]. We suggest that the type of immune responses induced by BCG or rBCG vaccines might be dependent on the adjuvants, vaccine types, immunization routes, and immunization doses used in these mouse models.

A further advantage of mouse models is their ease for genetic manipulation. Recently, several immunodeficient and gene knockout mouse models, including severe combined immune deficiency (SCID) mice [63], C3HeB/FeJ mice (model of liquefactive necrosis and necrotic granulomas) [64, 65], CBA/J IL-10(−/−) mice (mature, fibrotic *M. tuberculosis*-containing pulmonary granulomas) [66], C57BL/6 RAG(−/−) mice (small and diffuse lesions, with the majority of the lung retaining the typical lacy alveolar appearance of normal lung tissue) [67], C57BL/6 IL-17(−/−) mice (less densely packed granulomas with mononuclear cells) [68], and iNOS knockout mice (granulomas similar to those that form in humans) [69], have been used to study particular immune responses to mycobacterial infections. However, accumulating evidence shows that *M. tuberculosis* infection could induce neither caseous granuloma nor central necrosis in the most widely used mouse models (except for C3HeB/FeJ mice) [70, 71], which was entirely different to the pattern observed in humans and guinea pigs [30]. Moreover, some mouse models have disadvantages for studying various stages of TB progression in human pathologies, including granuloma formation, liquefaction, cavity formation, and hematogenous spread of the disease [30, 72].

#### 2.1.2. Guinea Pigs

**Figure 2:**
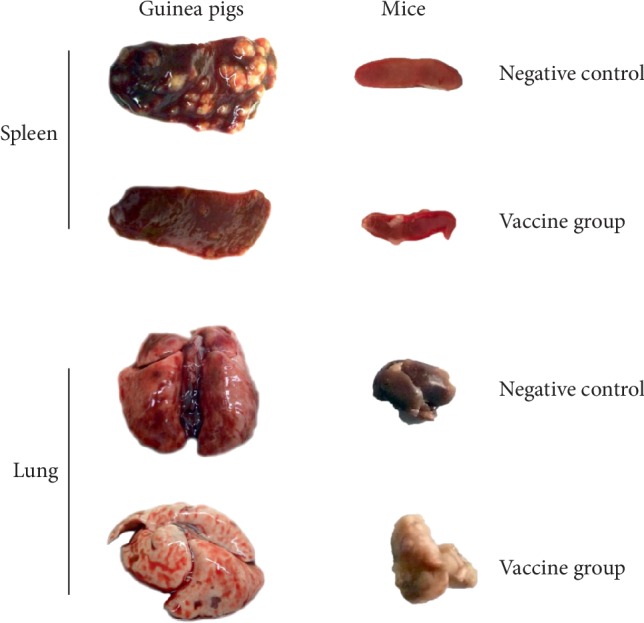
*Tubercles of spleen or lung collected from guinea pigs or mice infected with M. tuberculosis H37Rv strain*. BALB/c mice or guinea pigs were challenged with *M. tuberculosis* H37Rv strain (2 × 10^5^ CFUs or 5 × 10^3^ CFUs) to construct *M. tuberculosis* infected mouse or guinea pig TB model, respectively. After 3 days or 1 week, mice or guinea pigs were immunized intramuscularly three times at 2-weeks intervals with *M. tuberculosis* Ag85A/B chimeric DNA vaccine (vaccine group) or normal saline (negative control), respectively. Three weeks after last immunization, the mice or guinea pigs were sacrificed and their spleen and lung were collected to observe pathological lesions.

Guinea pigs were first used for mycobacterial infection studies as a very useful animal model for lymphocyte proliferation assays, and for evaluating dermal reactivity, new TB vaccine candidates, and the capacity of naturally transmitted multidrug-resistant *M. tuberculosis* because of their high susceptibility to *M. tuberculosis *infection via the airways [73, 74]. Pathological lesions that form on the inside and outside of the lungs of guinea pigs infected with *M. tuberculosis* have been widely studied, offering fundamental insight into pulmonary TB in guinea pigs [75, 76]. We and others reported that distinct gross pathological tubercles could be observed in the spleen of guinea pigs infected by *M. tuberculosis*, which were not observed in mice (Figure 2), whereas slight gross pathological tubercles could be observed in the lungs of both guinea pigs and mice (Figure 2) [35]. In particular, guinea pigs can develop classical granulomas that are structurally similar to those in humans, and Langerhans giant cells that are formed from macrophages and epithelioid cells after mycobacterium infection have been observed [10].

Furthermore, guinea pigs can be subsequently used to screen skin-test antigens, and to evaluate promising vaccines previously tested in a mouse model. A previous study also found that guinea pigs could be used as a long-term challenge model (with survival after 12 months) in assessment of TB vaccine efficacy [11]. Moreover, some vaccine candidates may be deemed to not be promising in the mouse model, but show satisfactory protection in guinea pigs as well as in humans. The immune responses of TB vaccines in guinea pigs have been studied by several methods such as antibody blocking, flow cytometry, bioassays, and microarray [12, 77], demonstrating that* M. tuberculosis* infection could initially activate responding T cells (mostly CD4 cells), which dramatically decreased in number 30 days after the infection and were gradually replaced by steadily increasing B cells and granulocytes [12]. Hiromatsu et al. [78] also found that immunization with the lipid antigens of mycobacteria induced a CD1-restricted immune response in guinea pigs. However, in comparison with the reagents available for other animal models, there are limited immunological reagents specific for this animal model available, which affects the utility of guinea pigs in the evaluation of TB vaccines. Therefore, there is an urgent need to develop specific immunological reagents for guinea pigs. Recently, a range of immunological reagents for guinea pigs have been developed, such as cloned guinea pig IL-17A cDNA and its recombinant protein [79], IL-10 cDNA and its recombinant protein [80], IL-4 cDNA [81], and IFN-*γ* cDNA [82].

#### 2.1.3. Rabbits

Rabbit models were first widely used in molecular immunology, and have since been gradually replaced by rodents such as mice. However, rabbits are still an excellent animal model for human TB vaccine research because of the similar manifestations of lesions (granulomas, liquefaction, and cavities) to those observed in humans [14, 15]. In particular, rabbit models have been extensively used to screen and evaluate potential vaccine candidates (such as BCG, *M. vaccae*, *M. microti* and subunit vaccines), and to determine the pathogenic factors and pathogenesis of cavities induced by* M. tuberculosis* H37Rv infection [15–20, 83]. In addition, Tsenova et al. [84] reported large confluent granulomas with expansive areas of central necrosis in the lungs of rabbits infected with *M. tuberculosis* HN878 strain. Furthermore, a recent review article reported that infection of *M. tuberculosis* Erdman, *M. tuberculosis* H37Rv, and *M. tuberculosis* CDC1551 in New Zealand white rabbits resulted in different pulmonary pathologies, which indicated that the virulence of *M. tuberculosis* strains will determine the lesion severity in rabbit models [6]. Some recent studies suggested that a BCG-challenge rabbit skin model could be a valuable method for selecting therapeutic agents [20] and evaluating TB vaccines [19]. Collectively, these data suggest that rabbit animal models can be used not only for H37Rv strain infection but also for infection of other strains such as* M. tuberculosis* HN878, *M. tuberculosis* Erdman, *M. tuberculosis* CDC1551, and *M. bovis*, which provides new insights into the selection of animal models for evaluation of TB vaccines. Although guinea pigs and rabbits have many desirable features as models for TB, the high cost, lack of reagents, difficult gene manipulation, and ethical considerations involving these models often preclude their suitability for long-term survival studies [85].

#### 2.1.4. Rats

Initially, it was widely believed that rats were insensitive to *M. tuberculosis* and that high doses of *M. tuberculosis* could neither kill rats nor induce typical TB pathological lesions and tuberculin susceptibility [86–88]. However, this view has changed. A large number of studies have found that rats are not only sensitive to *M. tuberculosis *but also show delayed hypersensitivity [89]. Compared with mice and guinea pigs, rats have several advantages as models, such as easy manipulation, relatively low cost, strong resistance, and easy blood collection [21]. Therefore, this animal model has been widely used in evaluating vaccine- or drug-induced resistance [22, 23], for determining anaerobic drug activity [90], estimating the efficacy of BCG vaccination [91], and discovering new TB drugs [21]. Previous studies have indicated that granulomatous lesions (which lack central necrosis) could be observed in the lungs, spleens, lymph nodes, and livers of *M. tuberculosis*-infected American cotton rats, Lewis rats, Wistar rats, and Sprague-Dawley rats [21, 24–26]. Interestingly, microelement deficiency (such as zinc) in the diet of rats could affect their humoral and cellular immune responses to BCG and ESAT-6/CFP-10 vaccination [92]. In addition to this limitation, similar to the situation with mice, the rat animal model has certain drawbacks, including not being able to mimic human pathological lung changes such as caseous necrosis, fibrosis, calcification, and cavitation.

### 2.2. Large Mammalian Models

Small mammalian animal models play important roles in the preliminary screening of new vaccine candidates. However, large mammalian animal models can effectively confirm the protective efficacy of the initially screened vaccines in systems that are more similar to humans. Additionally, compared to small mammalian models, large mammalian models are more like humans with respect to the genetic background and characteristics of immune responses; however, their disadvantages include few available commercial reagents, ethical limitations, high cost, and difficult genetic manipulation. As a rule, NHPs are always used in evaluating human TB vaccines, while other large mammalian animal models are usually used in testing animal TB vaccines.

#### 2.2.1. NHPs

NHPs are naturally susceptible to *M. tuberculosis* and their use in vaccine and drug development has a long history. The biggest differences between NHPs and other animal models are the close evolutionary relationship with humans [8] and the quite similar pathology as well as disease condition between NHPs and human beings [34], which indicates that the immune responses of NHP models are very similar to those of humans. In infected monkeys, widespread caseous necrosis and liquefaction of the caseous material with cavity formation have been observed [30], along with granulomas containing giant cells with a similar structure to that of human lung granulomas [34]. It is widely accepted that improved TB vaccines should be able to avoid interfering with TB diagnoses such as the tuberculin skin test (TST), interferon-gamma release assay (IGRA), and GeneXpert. As early as 1998, an additional test called the PRIMAGAM-IFN-*γ* test was developed to distinguish TB disease among NHPs by detecting cellular immune responses to a purified protein derivative antigen via the IFN-*γ* concentration in whole-blood samples [93]. However, the reliability of the IFN-*γ* response to tuberculin antigen in cynomolgus macaques remains controversial [94]. Based on the immunological characteristics mentioned above, NHPs have become one of the best animal models for screening and evaluating improved TB vaccines with no interference with the diagnosis of TB.

To date, a large number of novel TB vaccines have been evaluated in NHP animal models by gastrointestinal or respiratory mucosal delivery, and the delivery method of vaccination appears to have an influence on the protective efficacy of TB vaccines in these models. Jeyanathan et al. [95] reported that respiratory mucosal boost immunization with AdHu5Ag85A vaccine could improve the protective efficacy and enhance the antigen-specific IFN-*γ*^+^ T cell responses in BCG-primed NHPs. IFN-*γ* is a cytokine that is critical for innate and adaptive immunity against mycobacterial infection. Another study demonstrated that the BCG vaccine induced multifunctional CD4^+^ T-cells producing IFN-*γ* and TNF-*α*, which are associated with reduced disease pathology following subsequent *M. tuberculosis* infection [96]. However, a previous study suggested that IFN-*γ* production was not a reliable correlate of immune protection for vaccination protocols and might be more relevant for active disease [97].

Although primates are more similar to humans with respect to genetic background, pathogenesis, clinical symptoms, and the immune mechanisms of TB, they are generally only used to test vaccine candidates that have been identified as promising during pre-screening in small animal models, because the use of NHPs is limited by ethical concerns, high cost, time consumption, enormous variance among individuals, lack of necessity for new drug approval, and space requirements [8, 35]. An additional challenge in using NHPs to test new vaccine candidates for improved performance compared to BCG is the potential for variable responses after BCG vaccination, depending on which NHP species is used [98]. Moreover, it is difficult to obtain statistically significant results from NHP animal models because of the typical small sample sizes, and large individual and genetic differences involved.

#### 2.2.2. Cattle

Cattle are the natural host of *M. bovis*, and these infections are a major cause of economic losses and problems with animal welfare, along with a zoonotic risk, especially in developing countries [99]. BCG-vaccinated cattle always show a higher IFN-*γ* response, fewer lesions, and fewer bacilli per lesion [100, 101]. Compared with nonvaccinated cattle, the microscopically visible bacterial load, CD68^+^ macrophages, CD3^+^ T lymphocytes, WC1^+^*γδ* T cells, and CD4^+^ IFN-*γ*^+^ T cells were significantly reduced in lymph node granulomas [102, 103], and the expression of indoleamine 2,3-dioxygenase (considered to play an immunoregulatory role in the immune response to *M. tuberculosis*) was decreased in the granulomas of BCG-vaccinated cattle [27]. A more recent study showed that the protective efficacy of BCG in cattle gradually weakened, and the level of antigen-specific IFN-*γ* remained above baseline levels at two years post-vaccination [104]. Fortunately, this issue could be solved by BCG revaccination [105, 106], which supported the hypothesis that revaccination of BCG in humans might be effective in populations showing a negative response in the TST.

This model is also well-suited for the secondary screening of TB vaccines [28] and measuring elements of immune responses against mycobacteria [101]. Indeed, the cattle model has several advantages in TB vaccine research, including the fact that the clinical disease develops slowly, the granulomatous reactions and immune responses are similar to those observed in humans, and the possibility of vaccination involving neonatal calves [8, 29]. However, this model also has certain drawbacks, including high costs and absence of cavitations, which are seen in infected humans [30].

#### 2.2.3. Goats

Goats can be naturally infected by *Mycobacterium caprae* or *M. bovis *[107] and are used to evaluate vaccine efficacy by differences in body weight, gross pathology, and bacterial loads. Indeed, the typical caseous necrotizing granulomas with liquefactive necrosis and cavities can be observed in the goat model infected with *M. caprae* [31], which is similar to that of active TB in humans. Recently, some studies have demonstrated that BCG vaccination of goats afforded a certain degree of protection against experimental challenge with *M. bovis* or *M. caprae* by reducing the volume of gross lung lesions and the bacterial loads in pulmonary lymph nodes, and increasing weight gain [32, 108, 109]. Interestingly, we found that the differences in BCG vaccination route might have an impact on the resulting immunoresponse characteristics. Accumulating data show that positivity to the single intradermal test and IGRA was observed in subcutaneously, intramuscularly [108, 110], or intranasally [111] vaccinated kid goats, but not in orally vaccinated goats [112]. These studies also indicated that the goat could be a more feasible model than cattle and NHPs because of its smaller size, lower cost, and caseous granulomatous and cavitary lesions that resemble those found in human TB patients [32, 33].

### 2.3. Invertebrate Models

Although mammals have been widely used as experimental animal models in TB vaccine development, recent studies on *Mycobacterium marinum *infection in invertebrates have offered valuable insight into strategies for developing novel animal models. Furthermore, invertebrate models show several benefits in terms of resources, costs, technical convenience, and ethical acceptance.

#### 2.3.1. Zebrafish

Zebrafish (*Danio rerio*) can be naturally infected by *M. marinum* (a close relative of *M. tuberculosis* and the etiological agent of TB in humans), and is widely used as an animal model in vaccine research owing to its advantages of small size, easy reproduction, and low cost [36]. After infection by *M. marinum*, both adult zebrafish and larvae can form granulomas that are very similar to those observed in humans, and the innate and adaptive immune responses elicited against mycobacteria are composed of the same primary components found in humans [37–39]. In addition, the transparent characteristic of zebrafish larvae is also suitable for fluorescence imaging. Although zebrafishes are very different to humans in genetic terms, the above characteristics of this model have helped to bridge the gap between fish and humans. Data obtained from zebrafish studies have already shown that BCG vaccination, as well as DNA vaccination, can protect adult zebrafish from *M. marinum* infection by reducing both the mortality and bacterial counts in a manner dependent on the adaptive immune response and enhanced production of IFN-*γ* [38, 113]. In addition to its use for the preclinical screening of vaccines, the zebrafish model has been used in clarifying the mechanisms underlying granuloma formation [114]. Recently, several studies have indicated that this animal model provides a feasible tool for examining the mechanisms underlying reactivation in mycobacterial infections, and confirmed its suitability for the preclinical screening of TB vaccine candidates [38, 115–117]. However, a recent review indicated that the zebrafish model has significant differences in anatomy and physiology from those of humans [7], which warrant attention when using this animal model to evaluate TB vaccines.

#### 2.3.2. Fruit Fly

The fruit fly *Drosophila melanogaster* is also a good model for studying the innate immune responses to *M. marinum* infection, understanding the physiological consequences of such infection and the associated immune responses, along with anti-mycobacterial drug discovery [41]. As an animal model for studying host-pathogen interactions, *D. melanogaster* has significant advantages such as being easy to breed and handle, strong fecundity, short generation time, low cost, technical convenience, ethical acceptability, and genetic amenability [40, 41]. *D. melanogaster *can be infected by *M. marinum *through anesthetizing with CO_2_ and injection in the abdomen using an individually calibrated pulled glass needle, as characterized by widespread tissue damage and low bacterial loads [118]. Additionally, a previous study suggested that *M. marinum*-infected *D. melanogaster* showed a diabetes-like state with reduced levels of circulating insulin or increased turnover of activated Akt [119]. These pathological characteristics are similar to those found in the early stages of *M. marinum* infection in fish [42]. Thus, this model may be valuable in testing interactions between the pathogen and the host. However, the drawback of this model is that the fruit fly can only be used to study innate immunity because of the absence of adaptive immunity; therefore, experimental results still need to be confirmed in mammals.

#### 2.3.3. Amoeba

The amoeba species *Dictyostelium discoideum *is widely distributed in forest soil and can be infected by *M. marinum*, *M. tuberculosis*, and *M. bovis *[35, 120, 121]. *D. discoideum* has a haploid genome and a simple life cycle, which provides a genetically tractable single-cell model for studying conserved host–pathogen interactions [35]. As early as 2009, Soldati et al. [122] used *D. discoideum* as a genetically tractable host of *M. tuberculosis* and *M. marinum*, and discovered a conserved nonlytic spreading mechanism, in which pathogenic mycobacteria are ejected from the amoeba cell through the ejectosome, providing the opportunity for research into the spreading of tubercular mycobacteria infections in mammalian cells. Recently, the *D. discoideum* host model was developed to quantitatively monitor *M. marinum* growth, and to quantify the recruitment of host proteins to the bacterium-containing compartment [123, 124], assess the virulence of *M. marinum, *identify compounds inhibiting mycobacterial virulence [125], and recover new species of *Mycobacteria* from environmental and clinical specimens [126]. However, its application is limited, since it is single-cell model.

## 3. Lessons from Preclinical Experiments in Animal Models and Clinical Trials in Humans

The potential for a candidate vaccine to progress to the stage of efficacy evaluation in humans depends on the following main criteria: protection and safety in animal models, and safety as well as immunogenicity in Phase I/IIa clinical trials [127]. To date, BCG has been used as a “gold-standard” control vaccine to evaluate and compare the protection efficacy of new TB vaccine candidates in both preclinical animal models and clinical trials. Compared with BCG immunization in isolation, a good candidate TB vaccine should offer improvements in safety, immunogenicity, and protective efficacy (Table 2) [127]. A recent study showed that more than 85% of candidate drugs or vaccines that have passed preclinical testing failed in Phase I clinical trials [128]. Five well-known TB vaccine candidates that were successful in animal models but failed in clinical trials are recombinant BCG30 (rBCG30), AERAS-422, H1:LTK63, MVA85A, and SRL-172 (heat-killed *M. vaccae*) [3, 4, 129]. All five vaccines showed significant immunological protection and safety in animal models, but were terminated in clinical trials due to their poor protective efficacy and safety issues such as an antibiotic resistance gene in the case of rBCG30 [130], painful skin herpes for AERAS-422 [131], transient peripheral facial nerve palsies for H1:LTK63 [132], the absence of efficacy against TB for MVA85A [129], and technical issues for SRL-172 [133]. These data indicated that some negative results in terms of safety, immunogenicity, and protection efficacy were not observed in animal models. The following reasons were used to explain inconsistencies between animal preclinical data and clinical trials: (1) species differences between animal models and humans [134]; (2) differences in methodology between animal challenge experiments and natural infection in humans [134]; (3) fundamental differences in study schemes, protection efficacy definitions, and immunization strategies [135]; and (4) environmental differences such as environmental mycobacteria infection, BCG vaccination, and exposure level [135].

Although there are some barriers in translating the results of animal models to clinical trials, animal models are still the most effective tool for testing the safety and efficacy of TB vaccines, and they are still widely used by researchers worldwide. Herein, we take VPM1002 and MVA85A as examples to review the preclinical studies in the context of human clinical trials. VPM1002 is a recombinant BCG vaccine in which the urease C gene has been replaced by the listeriolysin O (*LLO*) gene [4]. VPM1002 can secrete LLO to accelerate the transport of BCG-derived antigens into the cytosol and promote the apoptosis and xerophagy of host cells *in vitro*. A growing number of studies have shown that the protective efficiency and/or safety of VMP1002 were improved compared with those of BCG tested in mice, guinea pigs, rabbits, and NHPs [4, 151–153]. After extensive preclinical development, the safety and immunogenicity of VPM1002, in comparison with BCG, have been successfully evaluated in two Phase I clinical trials conducted in adults and infants in South Africa (NCT01113281) and Germany (NCT00749034) [154]. The results showed that VPM1002 was safe and immunogenic, which is consistent with two subsequent Phase II clinical trials carried out in HIV-exposed/unexposed newborn infants in South Africa (NCT02391415) [149], and in adults in Germany (NCT02371447). At present, a Phase II/III clinical trial is being conducted in India to assess the efficacy and safety of VPM1002 (NCT03152903). In contrast, previous studies reported that MVA85A, a booster vaccine, showed protection efficacy in animal models, but failed to show better protective efficacy than BCG in Phase II clinical trials, which might be attributed to the fact that the clinical trial design did not include the same route of administration as that used in mice where efficacy was observed [155]. In summary, the above comparison of animal and human data of VPM1002 and MVA85A vaccines suggests that preclinical results in animal models will be more predictive and consistent if the study design is optimized to more closely reflect the targeted effects of vaccines in clinical trials.

## 4. Current Challenges and Future Opportunities

Over 22 new TB vaccines have passed through animal experiments to evaluation in clinical trials. However, development and further evaluations of four TB vaccine candidates were terminated owing to their disappointing results after Phase I or II clinical trials. Why are these hidden dangers not found early in animal models, but only later on in human volunteers? The answer to this question is rather complicated, but the main reasons may be the lack of suitable animal models for TB vaccines, experimental design defects, vaccine adverse events, and lack of a complete understanding of host immunity to TB [127]. Although animal models are indispensable tools for human TB vaccine research, no animal model can fully mimic the real situation of human TB disease. Therefore, the experimental results of animal models are only an indirect indication, and the protective effects of the vaccine need to be verified by clinical trials. The following sections will discuss the challenges and opportunities related to the use of animal models in TB vaccine development.

### 4.1. Interactions Between the Host and M. tuberculosis are Still Unclear

Previous studies have indicated that innate immunity and adaptive immunity play critical roles in controlling *M. tuberculosis* infection in humans [4, 156, 157]. Thus, to develop a suitable animal model for TB vaccine development, it is important to first understand the interplay between *M. tuberculosis *and the host. At the early stage of *M. tuberculosis *infection, *M. tuberculosis *can be first recognized and controlled by the innate immune cells such as macrophages, dendritic cells, neutrophils, and natural killer cells via pattern recognition receptors, phagocytosis, inflammasome activation, reactive oxygen species, autophagy, apoptosis, and production of nonspecific cytokines and chemokines [158–162]. However, *M. tuberculosis *has a special ability to escape from the immune surveillance of these innate immune cells [163]. Fortunately, this innate immunity “negligence” is overcome by adaptive immunity, especially cellular immunity. Class I or Class II major histocompatibility complex molecules bridge the gap between innate immunity and adaptive immunity by presenting *M. tuberculosis *antigens to CD4^+^ T cells such as Th1 and Th17 cells, or CD8^+^ T cells [164, 165]. A growing number of studies have suggested that Th1 and Th17 cells play a central role in host protection by secreting IFN-*γ*, TNF-*α*, and IL-17 [4, 166–172]. However, disappointingly, some vaccines have good immune protection and safety in animal models, but unexpected safety issues still arise in clinical trials. The reasons behind this variability in protective efficacy and safety are largely unknown, but we hypothesize that the differences could be due to differences in immune system biology between mice, NHPs, and humans.

### 4.2. Immunization Strategies Should Be Optimized Based on Different Animal Models

No clinical studies have established immunologic requirements for protection against TB. Despite endless immunologic observations, in the absence of controlled trials comparing immunologic responses among successful and unsuccessful vaccines (or controls), these observations do not meet established vaccine requirements. For this reason, there is increasing acknowledgement that it is problematic to extrapolate the findings from “successful” animal studies to clinical efficacy. To overcome this problem, immunization strategies should be optimized and improved. Currently, three immunization strategies are used in the development of new TB vaccine candidates, including an immunotherapy strategy, prime strategy, and BCG prime-boost strategy [173], and the TB vaccine candidates in clinical development can be divided into two groups: BCG replacements and BCG boosters [174]. This issue has also been explicitly addressed in recent World Health Organization position papers (https://apps.who.int/iris/handle/10665/273089) and the general conclusion is that the most efficient and cost-effective approach will be a BCG booster vaccine. BCG was used for TB prevention as early as 1921, and since then many clinical trials conducted worldwide have evaluated the efficacy of BCG in preventing TB. These tests have shown that BCG can continue to protect children from TB meningitis and disseminated TB [4]. However, a large number of studies have also shown that the protective effect of a BCG vaccine varies in different regions [175]. In addition, a large randomized controlled trial in Brazil showed that revaccinating BCG at adolescence did not improve the protective efficacy of BCG vaccination at birth [176]. Our recent study found that the main TB vaccine immunization strategy is BCG for primary immunization, followed by selection of appropriate subunit vaccines for boosting immunization [4], which is consistent with previous studies [177–179]. Therefore, we strongly recommend that further TB vaccine research should focus on a BCG booster vaccine, and animal models will provide an opportunity for conducting preclinical studies to demonstrate the protective efficacy of booster vaccines.

### 4.3. M. tuberculosis/HIV Co-Infection Has Become a Major Barrier for Fighting TB with Limited Appropriate Animal Models

LTBI is a condition characterized by a persistent immune response to stimulation by *M. tuberculosis* antigens without evidence of clinical manifestations of active TB [1]. However, when the immunity of patients with LTBI is weakened, the possibility of LTBI transforming into TB is greatly increased. Unfortunately, the decreased CD4^+^ T cell level of HIV patients provides an opportunity for LTBI reactivation to active TB [180]. According to published data, people infected with HIV are 16–27-times more likely to develop TB than healthy people, and HIV co-infection in individuals with LTBI enhances the risk of developing active TB from 10% over a lifetime to 10% per year [181]. Compounding this situation is the unique increased susceptibility of this population to any mycobacterial infection, which poses extraordinary challenges to the use of any live TB vaccines. Therefore, there is an urgent need to establish appropriate animal models to evaluate *M. tuberculosis*/HIV co-infection vaccines. Although HIV does not cause disease in rodents and NHPs, complementary mouse models and simian immunodeficiency virus (SIV; a retrovirus causing immunodeficiency similar to AIDS in Asian macaques) macaque models have been used for studies on *M. tuberculosis*/HIV co-infection [180]. Previous studies have reported two kinds of complementary mouse models, including a humanized mouse model and HIV transgenic mouse model. The first humanized mice were generated by reconstituting the immune system of immunodeficient mice using human hematopoietic progenitor cells (CD34^+^) from human cord blood [182]. The second one is a bone marrow, liver, thymic (BLT) mouse model in which NOD/scid-IL-2Rgammacnull mice are engrafted with human lymphoid tissue after CD34^+^ hematopoietic stem cell reconstitution [183]. These humanized mice gained human immunity by producing more proper humanized T cells, and have been used to evaluate new approaches for the prevention or treatment of HIV and/or *M. tuberculosis* infection [184–186]. An HIV transgenic mouse model was generated by incorporating the entire viral genome of HIV, which has been used to study the effect of *M. tuberculosis* infection on the induction of HIV gene expression [187]. In addition, a recent study found that NHPs could be infected by SIV, and SIV-infected macaques have been used as a model for AIDS and TB [188].

### 4.4. New Technologies and Tools Open New Avenues for the Use of Animal Models in TB Vaccine Research

The traditional use of animal models for vaccine research follows the conventional testing route through mouse models, then into guinea pigs or rabbits, which may be followed by testing in NHPs before moving to humans. However, traditional research methods are somewhat insufficient for the dynamic study of living experimental animals. Therefore, new technologies and tools are needed to observe the physiological, biochemical, and pathological changes in these living animal models, which will accelerate the development of TB vaccine research. Fortunately, new technologies and equipment have been employed in studies of animal models of TB and other diseases, including fluorescence microscopy to detect infection by *M. marinum*; robotic injection technology used in zebrafish embryos for high-throughput screening in disease models, which can greatly improve the injection efficiency and accuracy, and reduce errors caused by manual operation [189]; fluorescence-based methods for serial quantitative assessments of drug efficacy and toxicity [190]; photodynamic therapy technology in the treatment of localized mycobacterial infections such as pulmonary granulomas and cavities [191]; a three-dimensional granuloma model for studying bacterial-host interactions, drug-susceptibility, and resuscitation of dormant mycobacteria [192]; a small animal SPECT/PET/CT system for real-time dynamic observation of living animals, and for recording pathological changes, which can not only be more convenient and allow for objective evaluation of vaccine efficiency but also reduces the number of animals required as well as the impact of individual differences on vaccine evaluation [193]; and differentiating infected from vaccinated animals (DIVA) reagents in the skin test and IFN-*γ* assay in cattle and goats to differentiate TB-infected from vaccinated animals [194, 195]. Moreover, targeted genome editing technology has become a hot research area in animal models. Specifically, CRISPR/Cas9 technology, which greatly improves the efficiency of constructing gene-targeted animal models, has been widely used to construct genetically modified mouse models such as knockout/knockin models, and somatic cell genome-editing models [196].

### 4.5. Humanized Transgenic Animal Models Bring New Hope for TB Vaccine Research

TB vaccine candidates cannot be tested directly in human beings for ethical and safety reasons. Thus, humanized animal models could be useful to bridge the gap between preclinical and clinical studies, and to gain relevant insight into the determinants of TB vaccine development. Humanized mice (defined as mice engrafted with functional human genes, cells, or tissues) have become an essential tool in validating the results of infectious disease research in recent years because of their small size, easy access, low cost, clear genetic background, and easy manipulation. To date, large numbers of human cells or tissues have been engrafted in mouse models, including immune system components, hepatocytes, skin tissue, pancreatic islets, uterine endometrium, and neural cells [197]. Recently, some new humanized mouse models have been developed to identify potential TB or other vaccine candidates, including humanized NOD/shi-scid/*γ*_c_^null^ (NOG) mice [198], NOD/SCID/*γ*_c_^null^ (NSG) mice engrafted with human fetal liver and thymus tissues, and CD34^+^cells [199], DRAG mice (NSG mice transgenic for human DR4, RRID:IMSR_JAX:017914) [200], HSC-engrafted NSG mice [201], HLA-A2 transgenic NSG-BLT mice [201], and NOD.Cg-Prkdc^scid^ Il2rg^tm1Wjl^/SzJ mice [202]. As early as 2013, a BLT-humanized mouse model was developed to evaluate its feasibility as a model for experimental TB, demonstrating human T cells in the lung, liver, and spleen, and the formation of granuloma lesions with central necrosis and cholesterol crystals in the lung lesions [199]. Recently, a study compared the ability of the BCG vaccine to affect the immune response to infection with *M. tuberculosis* in C57BL/6 mice, Hartley guinea pigs, and humanized NOG mouse models, and the results indicated that the BCG vaccine could induce a human T cell response in the humanized NOG mouse model but not in the C57BL/6 mouse and Hartley guinea pig models [198]. Although the use of humanized animal models is limited by some shortcomings such as high cost, slow reproduction, and strict feeding conditions, the development of more novel humanized animal models will be important to create a crucial pre-clinical platform for evaluating the protective efficacy of TB vaccines, and for screening antigens, epitopes, and targets of TB vaccines.

### 4.6. Transmission of M. tuberculosis Infection among Different Animal Models Needs to be Considered

For thousands of years, animals have become important hosts of *M. tuberculosis* because of the close relationship between animals and the primary host human beings via coughing, sneezing, expectoration, or contaminated food [203–205]. We previously reported an infectious disease among 84 rhesus macaques at a Chinese zoo, and determined that the potential pathogen of this outbreak was likely *M. tuberculosis *[157]; the source of the infection may have been inhalation of airborne droplet nuclei from visitors infected with *M. tuberculosis*. In another study, Cadmus et al. [206] confirmed *M. tuberculosis* as the cause of pulmonary TB among livestock workers in southwestern Nigeria, and the mycobacteria pathogens likely originated from infected animals. *M. tuberculosis* can be transmitted not only between humans and animals but also between animals through similar transmission routes found in humans. Ramos et al. [207] reported that when W-Beijing *M. tuberculosis* HN878 infected or unchallenged minipigs were housed together, active *M. tuberculosis* could be identified in the pulmonary and thoracic lymph nodes from both infected and unchallenged minipigs. Furthermore, it is gradually becoming recognized that vaccination of domestic animals may be an alternative long-term strategy for controlling the transmission of TB [208].

## 5. Conclusion

Development of novel TB vaccines is urgently needed to fight TB disease. The animal models commonly used in human TB vaccine research include mice, guinea pigs, rabbits, rats, and NHPs, along with cattle, goats, and zebrafish in animal TB vaccine research. Along with recent developments in genetics, immunology, and molecular biology, some novel animal models have been introduced into TB vaccine research, such as fruit flies, amoeba, and humanized mouse models. The usage of humanized mouse models could overcome the disadvantages of NHPs and other animal models. In particular, the BLT humanized mouse has facilitated pioneering studies of TB pathogenesis, pathology, and vaccines [197, 199, 201, 209]. Although the cost of BLT mice is higher than that of small mammalian animal models such as mice, guinea pigs, and rabbits, the expense of BLT mice is considerably lower than that of NHPs. Therefore, the immune protective efficacy of TB vaccine candidates could be evaluated in humanized mouse models, which could shorten the research process and reduce costs [210]. Furthermore, the lack of protective biomarkers and understanding of the detailed host–pathogen relationship are the main obstacles hindering the evaluation of TB vaccines in animal models [211]. A recent review recommended some protective biomarkers such as survival, cytokines, pulmonary pathology, and bacterial load [6]. Therefore, these should be firmly integrated into future TB research, which will make the evaluation of a TB vaccine in animal models more diversified and objective, and will provide novel opportunities for the discovery of new TB vaccines.

## Figures and Tables

**Table 1:** Main animal models used in TB vaccine research.

Model kinds	Animal kinds	*Mycobacterium*	Granulomas (necrosis)	Advantages	Disadvantages	Main references
Small mammalian models	Mice	*M. tuberculosis*	No	Small and low cost	*M. tuberculosis* not a natural pathogen	[6–9]
				Genetically tractable	No granulomas formation	
				Mature immunological evaluation indexes		
				More abundant commercial reagents		
				Suitable for large-scale screening of vaccines and drugs		
	Guinea pigs	*M. tuberculosis*	Yes (+)	Highly susceptible to *M. tuberculosis*	Large and high cost	[10–13]
				Classical granulomas are similar to those in humans	Lack of reagents	
				Suitable for vaccine and drug studies	Genetic manipulation difficult	
	Rabbits	*M. tuberculosis*	Yes (+)	Highly susceptible to *M. tuberculosis*	Large and high cost	[14–20]
		*M. bovis*		Granulomas, liquefaction, and cavities are similar to those in humans	Lack of reagents	
				Suitable for vaccine and drug studies	Genetic manipulation difficult	
	Rats	*M. tuberculosis*	Yes (−)	Easy manipulation	Cannot mimic human lung pathological changes	[21–26]
				Low cost	Without caseous necrosis, fibrosis, calcification and cavitation	
				Easy blood collection		
				Suitable for vaccine and drug studies		

Large mammalian models	Cattle	*M. bovis*	Yes (±)	*M. bovis* is a natural pathogen	Large and high cost	[8, 27–30]
				Granulomatous reactions and immune responses are similar to those in humans	Genetic manipulation difficult	
				Secondary screening of TB vaccines		
				Availability of reagents		
	Goats	*M. caprae*	Yes (+)	*M. caprae* is a natural pathogen	Large and high cost	[31–33]
				Typical caseous necrotizing granulomas with liquefactive necrosis and cavities	Genetic manipulation difficult	
				Vaccination studies		
	Nonhuman primates	*M. tuberculosis*	Yes (+)	Susceptible to *M. tuberculosis*	Large and high cost	[8, 30, 34, 35]
				Availability of reagents	Space requirements	
				Disease pathology like human TB	Ethical concerns	
					Small sample size	

Invertebrate models	Zebrafish	*M. marinum*	Yes (−)	Disease progression and pathology like humans	Lack of cell lines	[7, 36–39]
				Genetically tractable	Anatomy and physiology are unlike humans	
				Suitable for large-scale screening of vaccines and drugs		
	Fruit flies	*M. marinum*	No	Innate immunity well conserved	Mycobacteria are not natural pathogens	[40–42]
				Wasting phenotype like human TB	No granulomas	
				Genetically tractable	No adaptive immunity	

*M. marinum*: *Mycobacterium marinum*, *M. tuberculosis*: *Mycobacterium tuberculosis*, *M. bovis*: *Mycobacterium tuberculosis*, *M. caprae*: *Mycobacterium caprae*, BCG: Bacille-Calmette–Guerin, TB: tuberculosis. +: Necrosis within granulomas, −: no necrosis within granulomas, *±*: granulomas in cattle animal model were staged (I–IV) based on cellular composition and the presence or absence of necrosis and peripheral fibrosis.

**Table 2:** Comparison of safety, immunogenicity, and protective efficacy of current TB vaccines and BCG in preclinical and clinical trials.

Status	Vaccine	Animal models or populations	Immunization route and dose^a^	Safety	Immunogenicity	Efficacy	Failure reasons	References or NCT No^b^
Preclinical	VV-tPA-85B	Mice	Unknown	Unknown	IFN-*γ* two times higher	Less	NA	[136]
	PcDNA3.1-Rv1769 or PcDNA3.1-Rv1772	BALB/c mice	50 *μ*g DNA, s.c.	Unknown	Induce long-lasting Th1-type responses and CD8^+^ T-cell response	Unknown	NA	[137]
	rBCGΔais1/zmp1	Guinea pig	5 × 10^4^ CFUs, s.c.	Safe	Stimulate high immunogenicity and strong memory immune responses	Improved BCG protection by 0.91 log10	NA	[138]
	BCGΔBCG1419c	BALB/c mice	2.5 × 10^3^ CFUs, s.c.	Safe	A better activation of specific T-lymphocytes population	Similar	NA	[139]
	rBCG: CysVac2	C57BL/6 mice	5 × 10^5^ CFUs, s.c.	Safe	Enhanced antigen-specific CD4^+^ T cell priming	Similar	NA	[140]
	rBCG-CMX	BALB/c mice	1 × 10^6^ CFUs, s.c.	Safe	Higher levels of CD4^+^IL^−^17^+^ and CD4^+^IFN-*γ*^+^T cells	Improved	NA	[58]
	ChAdOx1.PPE15	C57BL/6 and BALB/c mice	1 × 10^8^ infectious units, i.n. or i.d.	Safe	More lung parenchymal CD4^+^ and CD8^+^ CXCR3^+^ KLRG1^−^ T cells	Improved BCG protection by 0.52 log10 in C57bl/6 mice	NA	[46]
	BER ^opt^	BALB/c mice	100 *μ*g DNA, i.m./EP	Safe	Induce surprisingly high frequencies of Ag85B tetramer^+^ CD8^+^ T cells and IFN-*γ*-secreting CD8^+^ T cells	Similar	NA	[141]
	MtbΔlpqS	Guinea pigs	50–100 CFUs, respiratory	Safe	Expression of IFN-*γ* and IL-10 was lower in the lungs	Superior protection than BCG by 1 log10	NA	[142]

Phase I	Ad5Ag85A	Both BCG-naïve and previously BCG-immunized healthy adults (24 participants)	1 × 10^8^ PFUs (low dose), 1 × 10^9^ PFUs (high dose), i.m.	Well tolerated, no serious adverse effects	Markedly increased antigen-specific responses of both polyfunctional CD4^+^ and CD8^+^ T cells	NA	NA	NCT00800670, [143]
	ChAdOx1.85A	Healthy BCG-vaccinated adults (42 participants)	Starter group (*n* = 6), 5 × 10^9^ vp; Group A (*n* = 12), 2.5 × 10^10^ vp, Group B (*n* = 12), 2.5 × 10^10^ vp + 1 × 10^8^ PFU of MVA85A, Group C (*n* = 12), 2.5 × 10^10^ vp + 1 × 10^8^ PFU of MVA85A, i.m.	No data	No data	NA	NA	NCT01829490
	H1:LTK63	BCG vaccine-naïve healthy subjects (9 participants)	100 *µ*g H1 mixed with 30 *µ*g LTK63, i.n.	Two volunteers experienced transient peripheral facial nerve palsies	No data	NA	Transient facial paralysis	NCT00440544, [132]
	rBCG30	PPD^−^/HIV^−^ healthy adults (35 participants)	5 × 10^5^ CFUs (*n* = 35), i.d.	Well tolerated, no serious adverse effects	Significantly improved antigen-specific T cell expansion capacity, IFN-*γ* secretion, and phenotypes of CD4^+^ and CD8^+^ T cells	NA	Potential danger from antibiotic resistance gene	[130, 144]
	AERAS-422	HIV negative BCG naïve healthy adults (24 participants)	>10^5^–<10^6^ CFUs (low dose, *n* = 8),10^6^–10^7^ CFUs (high dose, *n* = 8), 1–8 × 10^5^ CFUs (BCG, *n* = 8) i.d.	Unexpectedly, VZV reactivation (zoster) occurred in 2/8 volunteers	Stronger immune response in CD8^+^ T cells	NA	Painful skin herpes	[131, 145]

Phase IIa	H56:IC31	HIV-negative, BCG-vaccinated adults (98 participants)	3 dose levels: 5, 15, and 50 *µ*g of H56 antigen with 500 nmol IC31, i.m.	Well tolerated, no serious adverse effects	Induced functional profiles of antigen-specific CD4 T cells	NA	NA	NCT01865487 [146]
	ID93 + GLA-SE	HIV negative TB patients (60 participants)	2 or 10 mcg ID93 + 2 mcg GLA-SE Vaccine (low dose) and 2 mcg ID93 + 5 mcg GLA-SE Vaccine (low dose), i.m.	No data	No data	NA	NA	NCT02465216
	TB/FLU-04L	Unknown	Unknown	Ongoing	Ongoing	NA	NA	[1]
	MTBVAC	HIV unexposed, BCG naïve newborns (99 participants)	2.5 × 10^4^ CFUs (intermediate dose, *n* = 25), 2.5 × 10^5^ CFUs (high dose, *n* = 25), 2.5 × 10^6^ CFUs (highest dose, *n* = 25), BCG 2.5 × 10^5^ CFUs (*n* = 24), i.d.	Well tolerated, no serious adverse effects	A greater frequency of polyfunctional CD4^+^ central memory T cells	NA	NA	[147], NCT03536117

Phase IIb	DAR-901 booster	BCG-vaccinated, IGRA-negative healthy adolescents (650 participants)	0.1 ml intradermal injection of 1 mg DAR-901 (*n* = 325), saline control (*n* = 325)	Ongoing	Ongoing	NA	NA	NCT02712424
	M72/AS01E	TB-naïve adults (*n* = 80), adults previously treated for TB (*n* = 49), and adults who have completed the intensive phase of TB treatment (*n* = 13), total 142 participants	Two doses of M72/AS01E (*n* = 71) or placebo (*n* = 71) and followed-up until six months post-dose 2	Unexplained local reactions were observed, and further recruitment and vaccination in this study was discontinued	Stronger CD4^+^ T cell immune responses, rather than CD8^+^ T cell responses	NA	NA	NCT01424501 [148]
	MVA85A	BCG-vaccinated, HIV-negative healthy infants (2797 participants)	1 × 10^8^ PFUs MVA85A (*n* = 1399), placebo control (*n* = 1398), i.d.	Well tolerated, no serious adverse effects	Induced highly durable Th1 responses	Failed to improve BCG efficacy in infants	Unreasonable design, inappropriate study subjects, and too short observation time	NCT00953927 [129]

Phase III	VPM1002	Pulmonary TB patients completed ATT and declared cured (2000 participants)	Single dose of VPM1002 (*n* = 1000), placebo control (*n* = 1000), i.d.	Safe, no serious adverse events	Stimulated multifunctional T cells producing IFN-*γ* or B cells producing antibodies	Ongoing	NA	[149], NCT03152903
	Vaccae™	Cases whose skin tests of PPD are strongly positive (10000 participants)	One vial of Vaccae diluted with 1.0 ml sterile water, i.m., once every 2 weeks, 6 times totally	Safe and well-tolerated, no serious adverse events	Improved immunity and phagocytosis, and reduced pathological damage	TB incidence and degree of pathological changes of experimental group are lower than those of control group	NA	[150], NCT01979900
	MIP/Mw	Cat II PTB patients (1020 participants)	1 × 10^9^ heat killed organisms followed 6 months later with a 2nd dose of 5 × 10^8^ organisms	Safe, no serious adverse events	Higher IL-2 and IFN-gamma secretion	Significantly higher number of patients in the *MIP* group showing sputum culture conversion as early as 4 weeks after initiation of therapy	NA	NCT00265226, [2]
	SRL-172 (*M. vaccae*)	BCG-vaccinated, HIV-infected patients with CD4 cell counts of at least 200 cells/ml (1962 participants)	0.1 ml *M. vaccae* (SRL-172, 1 mg, 10^9^ CFUs, *n* = 983), placebo control (*n* = 979), i.d.	Safe, no adverse effect, and no increase in the rate of serious adverse events	SRL-172 immunization boostsIFN-*γ* and LPA responses to MV sonicate, and antibody responses to LAM	Protection was significant for the secondary endpoint of definite TB but not for probable TB	Technical reasons related to the method of production	NCT00052195 [3, 133]

^a^s.c., i.n., i.d., i.m./EP: animal models were immunized subcutaneously, intranasally, intradermally, or by intramuscular electroporation, respectively.

^b^ClinicalTrials.gov Identifier NCT number.

NA, not available; No data, data cannot be obtained from the public database.
